# First Chikungunya Outbreak in Suriname; Clinical and Epidemiological Features

**DOI:** 10.1371/journal.pntd.0004625

**Published:** 2016-04-15

**Authors:** Farah T. van Genderen, Ingrid Krishnadath, Rachel Sno, Meritha G. Grunberg, Wilco Zijlmans, Malti R. Adhin

**Affiliations:** 1 Department of Biochemistry, Faculty of Medical Sciences, Anton de Kom Universiteit van Suriname, Paramaribo, Suriname; 2 Department of Public Health, Faculty of Medical Sciences, Anton de Kom Universiteit van Suriname, Paramaribo, Suriname; 3 Department of Biochemistry, ‘Prof. Dr. Paul C. Flu’ Institute for Biomedical Sciences, Paramaribo, Suriname; 4 Scientific Research Center, Academic Hospital Paramaribo, Paramaribo, Suriname; Aix Marseille University, Institute of Research for Development, and EHESP School of Public Health, FRANCE

## Abstract

**Background:**

In June 2014, Suriname faced the first Chikungunya outbreak. Since international reports mostly focus on hospitalized patients, the least affected group, a study was conducted to describe clinical characteristics of mainly outpatients including children. In addition, the cumulative incidence of this first epidemic was investigated.

**Methodology:**

During August and September 2014, clinically suspected Chikungunya cases were included in a prospective follow-up study. Blood specimens were collected and tested for viral RNA presence. Detailed clinical information was gathered through multiple telephone surveys until day 180. In addition, a three stage household-based cluster with a cross-sectional design was conducted in October, December 2014 and March 2015 to assess the cumulative incidence.

**Principal Findings:**

Sixty-eight percent of symptomatic patients tested positive for Chikungunya virus (CHIKV). Arthralgia and pain in the fingers were distinctive for viremic CHIKV infected patients. Viremic CHIKV infected children (≤12 years) characteristically displayed headache and vomiting, while arthralgia was less common at onset. The disease was cleared within seven days by 20% of the patients, while 22% of the viremic CHIKV infected patients, mostly women and elderly reported persistent arthralgia at day 180. The extrapolated cumulative CHIKV incidence in Paramaribo was 249 cases per 1000 persons, based on CHIKV self-reported cases in 53.1% of the households and 90.4% IgG detected in a subset of self-reported CHIKV+ persons. CHIKV peaked in the dry season and a drastic decrease in CHIKV patients coincided with a governmental campaign to reduce mosquito breeding sites.

**Conclusions/Significance:**

This study revealed that persistent arthralgia was a concern, but occurred less frequently in an outpatient setting. The data support a less severe pathological outcome for Caribbean CHIKV infections. This study augments incidence data available for first outbreaks in the region and showed that actions undertaken at the national level to mount responses may have positively impacted containment of this CHIKV outbreak.

## Author Summary

Chikungunya virus is transmitted to humans by mosquito bites and causes fever and joint pain. Chikungunya was first detected in Africa, but recently became a worldwide concern with outbreaks in many (sub)-tropical countries. We report the characteristics of the first outbreak in Suriname (2014–2015). Mainly non-hospitalized patients were followed-up to study the clinical manifestations and course of the disease, after presentation in the respective clinics with the standard Chikungunya symptoms (fever and arthralgia). Twenty percent of follow-up patients could clear the disease within one week and 22% (mostly women and elderly) still had complaints about arthralgia up to 6 months after infection. This is consistent with the assumption that Caribbean Chikungunya viral infection has a less severe pathological outcome. Furthermore, more insight was gained into the symptomatology of children (≤12 years). In addition, house-to-house surveys in Paramaribo were carried out to identify suspected cases to assess the incidence. Almost 25% of the survey participants experienced symptoms consistent with Chikungunya during the nine months spanning the investigation. The launch of a governmental campaign to eliminate mosquito breeding sites coincided with a sharp decline of Chikungunya cases, suggesting that such measures may be important in the containment of future CHIKV outbreaks.

## Introduction

Chikungunya fever is caused by a classical arbovirus (genus *Alphavirus*, family *Togaviridae*), which is transmitted to humans primarily through *Aedes aegypti* and *Aedes albopictus* mosquitoes [1]. Acute onset of fever and polyarthralgia, mainly affecting the extremities (wrists, ankles, phalanges), are the primary reported clinical characteristics [2, 3]. Joint pain is often severe [4] and arthralgia may persist for weeks to years [5, 6]. Other reported symptoms include rash, headache and back pain [1, 7]. Despite the low hospitalization rate of Chikungunya patients (0.3% during the outbreak in La Reunion in 2005–2006 [8]), at present more is known about the clinical presentation and outcome (*i*.*e*. recovery or persistent pain) of hospitalized patients during outbreaks.

Chikungunya is endemic in tropical Africa, South-East Asia and on the Indian subcontinent [1, 3].

In recent years, outbreaks have been appearing outside the endemic zone probably due to increased global air travel and seaborne trade [9–12] and Chikungunya has recently emerged as a major public health concern in the Caribbean Region [13]. In December 2013, the first Chikungunya virus (CHIKV) infections were reported among non-travelers on the Caribbean island of Saint Martin [14]. Since then, the virus has spread rapidly into the Caribbean region and neighboring countries [15]. The Chikungunya viruses studied from this region belonged to the Asian genotype [16]. Recently, it was demonstrated that this strain causes a less severe pathological outcome compared to the East Central South African (ECSA) genotype [17].

In June 2014, the first locally acquired case of Chikungunya was reported in Suriname [18]. In this period, no other outbreaks of vector-borne diseases (e.g. yellow fever, malaria, leptospirosis) were recorded by the Bureau of Public Health, BOG (BOG Annual Report). A study was initiated with two objectives, firstly to assess the manifestation and course of Chikungunya infection following acute illness in a naïve population, and secondly to determine the cumulative incidence of the Chikungunya outbreak in Suriname. To assess the extent and severity of the symptoms during this first outbreak, patients with suspected CHIKV diagnosis were enrolled in the period between August and September 2014 in three different outpatient settings. CHIKV presence was determined with Real-Time reverse transcription-polymerase chain reaction (RT-rPCR). Detailed clinical information from Chikungunya infected patients was collected and the symptom development was recorded through multiple telephone interviews until 6 months after onset.

To determine the cumulative incidence of this first Chikungunya outbreak in Suriname, a community surveillance consisting of three successive household-based cluster investigations was conducted in the capital in October 2014, December 2014 and March 2015, gathering information on 1169 households with 4842 participants. The validity of self-reported CHIKV infections was crosschecked with serological analysis.

To our knowledge, the study is unique in describing in detail the clinical evolution of autochthonous CHIKV outpatients in a Caribbean country. Furthermore, clinical follow-up was conducted for six months for almost one hundred viremic CHIKV infected patients including 10 children (2–12 years). We therefore also add to the records internationally available for clinical manifestation of Chikungunya in children. The prospective follow-up study was complemented with cluster-based cross-sectional data, adding valuable data to the international Chikungunya outbreak information.

## Materials and Methods

### Setting

Suriname is a tropical country, located along the North Coast of South-America, bordering Brazil to the south, Guyana to the west and French Guiana to the east. Approximately 80% of Surname is covered by tropical rainforest. Suriname has a highly multiethnic population of nearly 550,000, most of whom live in the coastal area in and around the capital Paramaribo [19]. This study took place in Paramaribo and in Commewijne, a rural district adjacent to Paramaribo.

### Manifestation and course of CHIKV symptoms

#### Study design

A prospective cohort study during the first CHIKV outbreak in Suriname was carried out from August 2014 to April 2015. Eligible patients were enrolled in two general practices: a private practice located in Paramaribo and a governmental practice in a rural area (Commewijne). To acquire a better representation of the population, patients were also included on Sundays in the emergency clinic of the Academic Hospital of Paramaribo in the capital, where the whole community seeks medical care due to closed private and governmental practices. Patients were included in the study if they presented with a sudden onset of fever (>38.5°C) and arthralgia/arthritis not explained by any other medical condition (*i*.*e*. clinically suspected cases). Patients with respiratory symptoms and patients with an onset of more than 6 days were excluded. A short questionnaire was filled in by physicians to collect information on demographic characteristics including age and gender, presence of arthritis and periarticular edema. Furthermore, joint pain intensity was evaluated using a numerical rating scale (0–10 NRS score). By assigning a score between 0 and 10, the pain intensity of the patient was categorized as mild (score between 1 and 3), moderate (score between 4 and 6) or severe (score between 7 and 10) [20].

Consenting participants were interviewed by telephone by three trained investigators. The first interview followed as soon as possible after inclusion in the study to minimize recall bias. For persons aged less than 15 years, parents or legal guardians were interviewed. A questionnaire was used to gather information about date of onset, history of travel, residence and CHIKV symptoms such as musculoskeletal pain, fatigue, headache and skin rash. As most symptoms were collected retrospectively, they were defined only in general terms as presence/absence without a severity indication. The location of joint pain was also recorded. Children below 5 years old were only followed-up for symptoms observed by parents as the presence/absence of fever, arthralgia and rash. Patients with a positive RT-rPCR were regarded as confirmed cases of a CHIKV infection in the viremic stage. Patients with a negative RT-rPCR were designated as CHIKV- patients, although this category can also include non viremic CHIKV infected patients. Clinical data for all patients were collected from the day of onset (defined as day 0, D0) until 7 days (D7) after onset. Viremic CHIKV infected patients were also interviewed in the acute stage on day 14 (D14); in the post-acute stage on day 30 (D30) and day 90 (D90) in order to monitor the trend of clinical manifestations. Potential relapsing was assessed at D90 and day 180 (D180) and was defined as joint pain that lasted >1 day after a symptom-free period of >1 month [21]. The final outcome, complete clinical recovery or persistent arthralgia *i*.*e*. intermittent or continuous joint pain [21], was evaluated in the chronic stage on day 180 (D180).

#### Nucleic acid extraction and RT-rPCR testing

Serum samples were collected from all clinically suspected cases on the day of consultation. Samples were aliquoted and stored at -80°C until use. Viral RNA from 140 μL of serum was extracted with the QIAamp viral RNA kit (Qiagen Inc., Benelux B.V., Venlo, the Netherlands) according to the manufacturer’s instruction. CHIKV RNA was determined by RT-rPCR targeting the *E1* gene, according to a protocol adapted from Pastorino *et al*. [22]. One-step RT-rPCR reactions were run on a StepOnePlus Real-Time PCR system (Applied Biosystems, Foster City, California, USA) in a 25 μL reaction volume using 5 μL RNA and AgPath-ID one-step RT-PCR reagents (Applied Biosystems, Foster City, California, USA). The PCR program consisted of a RT reaction of 15 min at 50°C, 2 min at 95°C, followed by 50 cycles of 10 sec at 95°C, 30 sec at 60°C and 30 sec at 40°C. Nuclease-free water (duplo) was included in every run as a negative control (Ct = 0) and a positive control was used to monitor inter-run variability. Sample quality, RNA extraction and inhibitors of the PCR reaction were monitored with RNAse P as internal RNA control.

### Community-based surveillance

#### Study design

The study was a three-stage survey of household-based clusters with a cross-sectional design. The household-based clusters were not connected to the index cases enrolled in the prospective survey. Investigations were conducted in October 2014, December 2014 and March 2015 in the capital Paramaribo to identify and enlist suspected CHIKV cases. A self-reported CHIKV suspected case was defined as a patient with acute severe or immobilizing joint pain with or without fever/with or without rash, since July 2014. Assuming a prevalence of 50% in a household and a confidence interval of 95%, a minimum sample size of 385 households was required. For each survey, a sample frame was generated for six sections based on all the streets in Paramaribo and the households were randomly selected for each section.

Consenting participants needed to be at least 18 years. Polls were conducted by trained field workers with standardized questionnaires to collect anonymous data on gender, date of onset, treatment seeking behavior and the use of insect repellent within households. No data was collected on hospitalization or mortality. The field work for each survey was finalized within two days.

In week 40 (October 2014), a governmental campaign was launched in which mosquito breeding places were eliminated, bulky waste was collected and different neighborhoods were sprayed with insecticides.

#### Serological testing

In order to assess the validity of the self-reported CHIKV infections, serological testing was performed for 4% of the respective participants. Serum samples were collected from 52 randomly selected persons with a self-reported CHIKV infection for the analysis of IgG and IgM anti-chikungunya antibodies. Samples were stored at -20°C until transport on dry ice to RIVM (National Institute for Public Health and Environment) in the Netherlands for testing. The anti-chikungunya IgM and IgG indirect immunofluorescence (IFA) assay from EUROIMMUN (Lübeck, Germany) was used to detect IgM and IgG antibodies.

### Ethics statement

All participants provided oral informed consent. Acquired informed consent was registered prior to inclusion either by the attending physician on the short questionnaire in the clinical setting or by the trained pollster in the community surveillance. Participants, whose blood specimens were shipped abroad for testing, provided written informed consent. The national ethics committee within the Ministry of Health approved both studies (VG018-14 and VG008-15).

### Statistical analysis

All reported signs and symptoms are presented descriptively, namely the presence/absence of arthritis, periarticular edema, arthralgia, fever, myalgia, skin rash, itching, headache, back pain, eye pain, vomiting, nausea, fatigue, asthenia and joint pain intensity and location. The Chi-square test was used to compare characteristics and symptoms of the participants. To evaluate differences in age distribution between the viremic CHIKV infected and the CHIKV- group, the Mann-Whitney *U* test was used.

The Statistical Packages for Social Sciences (SPSS 21.0) were used for analysis excluding observations with missing data. Statistical significance was set at p = 0.05.

## Results

### Manifestation and course of CHIKV symptoms

#### Participants

**Fig 1:**
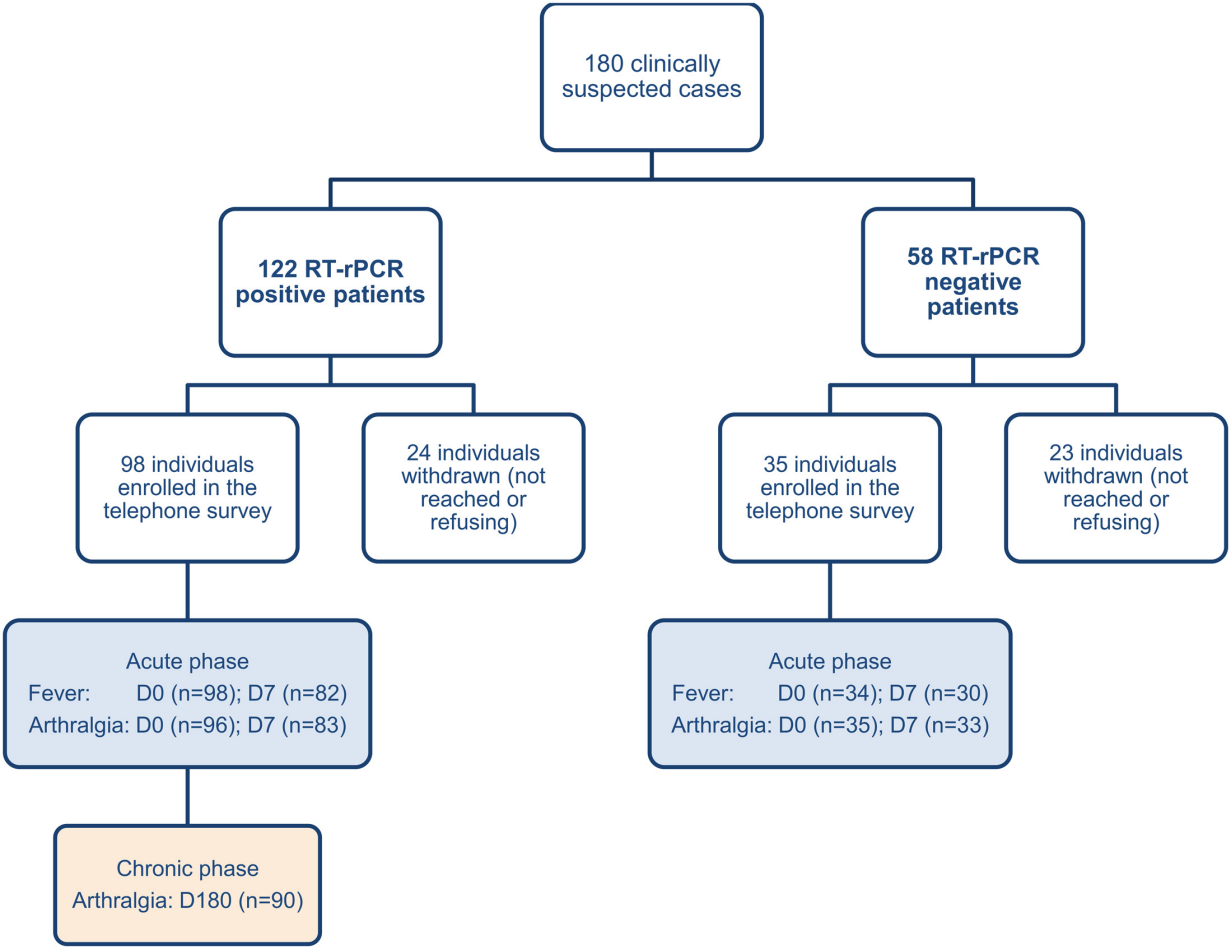
Study participation profile of the Chikungunya cohort for the prospective study.

180 clinically suspected patients were included in our study. All patients were residing in Suriname, except for one patient from the Netherlands. 56.7% of the patients were included in Paramaribo, 15.6% in Commewijne and 27.8% by the emergency clinic of the Academic Hospital of Paramaribo. Four patients travelled abroad in the month before inclusion: French Guiana (n = 2), USA and Columbia. Results from interviews conducted by telephone were available for 60.3% CHIKV- patients and 80.3% viremic CHIKV infected patients (Fig 1).

During the crosscheck of date of onset in these interviews, six viremic CHIKV infected patients mentioned to have onset of some symptoms of more than 6 days before inclusion. Four patients (2.2%) were hospitalized, one CHIKV- patient on day 5 and three viremic CHIKV infected patients, at day 1, day 4 and day 7, respectively. Two of the hospitalized viremic CHIKV infected patients died in the months after acute illness; a 52-years-old woman and the eldest male patient (82 years) who was hospitalized at inclusion. Both of them had severe underlying illnesses.

The median age of the study population was 32 years (range 2–82 years old). A total of 13 children (2–12 years old) were included. The gender and age distribution did not follow the general Surinamese population [19], as the male/female ratio (m/f: 0.48 *vs* 1.0) and persons less than 15 years old (9.4% *vs* 27.6%) were lower in our study population. The representation of ethnic groups was in line with the population distribution [19] with the exception of the Maroons, who were underrepresented (3.9% *vs* 21.7%).

Clinically suspected acute CHIKV infection was confirmed with RT-rPCR in 68% of the cases (viremic CHIKV infected patients), while 58 patients were negative with RT-rPCR (CHIKV- cases) (Fig 1).

#### Comparison of symptoms between viremic CHIKV infected and CHIKV- patients

The differences in manifestations in viremic CHIKV infected and CHIKV- patients are represented in Table 1. Analysis of the viremic CHIKV infected and CHIKV- patients at inclusion revealed no differences in sex ratio (m/f) and age distribution. Furthermore, at inclusion, the median score of pain intensity (median NRS score = 8) and the frequency of arthritis and periarticular edema were similar for viremic CHIKV infected and CHIKV- patients (Table 1). At disease onset, viremic CHIKV infected persons had more frequent headache, arthralgia and pain in the fingers. Moreover, arthralgia was more frequently reported at D1 and D2 in viremic CHIKV infected patients, while fever at days 4 and 5, myalgia at days 3 and 4, skin rash at days 5 and 6, itching at day 6 and eye pain at days 6 and 7 were reported less frequently (Table 1).

**Table 1:** Characteristics of viremic CHIKV infected and CHIKV- patients^a^.

	RT-rPCR positive patients n (%)	RT-rPCR negative patients n (%)	p-Value
**Gender**			
**Male**	39/121 **(32.2)**	19/58 **(32.8)**	0.94
**Female**	82/121 **(67.8)**	39/58 **(67.2)**	
**Age**			
**≤15**	18/118 **(15.2)**	1/57 **(1.7)**	
**16–30**	32/118 **(27.1)**	26/57 **(45.6)**	
**31–45**	32/118 **(27.1)**	18/57 **(31.6)**	
**46+**	36/118 **(30.5)**	12/57 **(21.1)**	
**Median (95% CI)**	33.5 **(3.0–73.2)**	31.0 **(14.3–63.6)**	0.66
**Pain intensity (NRS score)**			0.30
**4**	2/65 **(3.1)**	0/26 **(0.0)**	
**5**	5/65 **(7.7)**	0/26 **(0.0)**	
**6**	12/65 **(18.5)**	2/26 **(7.7)**	
**7**	6/65 **(9.2)**	5/26 **(19.2)**	
**8**	20/65 **(30.8)**	12/26 **(46.2)**	
**9**	7/65 **(10.8)**	2/26 **(7.7)**	
**10**	13/65 **(20.0)**	5/26 **(19.2)**	
**Condition registered at inclusion**			
**Periarticular edema**	13/51 **(25.5)**	6/19 **(31.6)**	0.61
**Arthritis**	29/81 **(35.8)**	9/30 **(30.0)**	0.57
**Symptoms at onset**^b^			
**Arthralgia**	81/96 **(84.4)**	23/35 **(65.7)**	0.019
**Headache**	49/90 **(54.4)**	12/35 **(34.3)**	0.043
**Pain fingers**	47/92 **(51.1)**	11/35 **(31.4)**	0.047
**Symptoms D1-D7**^b^			
**Arthralgia day 1**	87/95 **(91.6)**	24/35 **(68.6)**	0.001
**Arthralgia day 2**	83/95 **(87.4)**	25/35 **(71.4)**	0.032
**Fever day 4**	26/95 **(27.4)**	17/33 **(51.5)**	0.011
**Fever day 5**	17/90 **(18.9)**	15/33 **(45.5)**	0.003
**Myalgia day 3**	36/92 **(39.1)**	22/35 **(62.9)**	0.016
**Myalgia day 4**	32/91 **(35.2)**	20/35 **(57.1)**	0.025
**Skin rash day 5**	27/88 **(30.7)**	18/34 **(52.9)**	0.022
**Skin rash day 6**	21/83 **(25.3)**	18/33 **(54.5)**	0.003
**Itching day 6**	15/76 **(19.7)**	13/33 **(39.7)**	0.031
**Eye pain day 6**	5/77 **(6.5)**	7/34 **(20.6)**	0.027
**Eye pain day 7**	4/74 **(5.4)**	6/33 **(18.2)**	0.036

^a^ n represents the actual number of reached patients for each category.

^b^ Only the symptoms with significant differences between viremic CHIKV infected and CHIKV- patients are represented.

#### Clinical profile of viremic CHIKV infected patients at inclusion

The pain intensity was severe at inclusion for 70.8% patients (NRS score between 7 and 10) and moderate for 29.2% patients (NRS score between 4 and 6). At consultation, 35.8% of the viremic CHIKV infected patients had arthritis and 25.5% had periarticular edema (Table 1).

**Fig 2:**
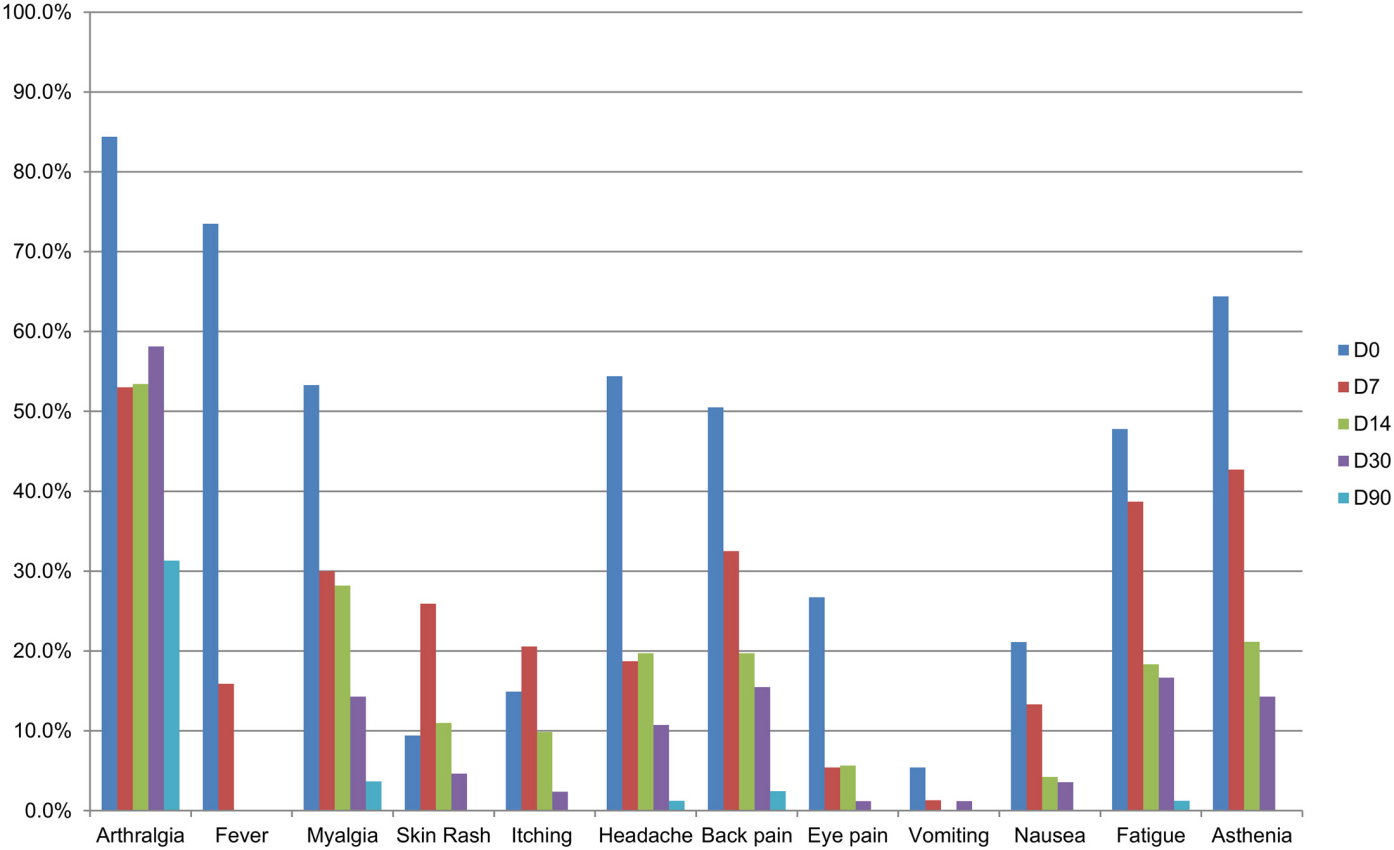
Clinical symptoms of viremic CHIKV infected patients at onset, day 7, 14, 30 and 90. Data collected from the viremic CHIKV infected individuals enrolled in the telephone survey. Actual number of patients at each time point per symptom: 1) D0: fever (n = 98), arthralgia and rash (n = 96), back pain and vomiting (n = 93), myalgia (n = 92), itching (n = 87) and other symptoms (n = 90); 2) D7: arthralgia (n = 83), fever (n = 82), rash (n = 81), myalgia (n = 80), vomiting (n = 78), back pain (n = 77), eye pain (n = 74), itching (n = 73) and other symptoms (n = 75); 3) D14: arthralgia (n = 73), rash (n = 72) and other symptoms (n = 71); 4) D30: arthralgia and rash (n = 86) and other symptoms (n = 84), and 5) D90: rash (n = 86), itching (n = 84), arthralgia (n = 83) and other symptoms (n = 82). Presence of fever was only registered until 7 days after infection.

Amongst the viremic CHIKV infected patients, general symptoms at onset most often recorded in descending order were arthralgia (84.4%), fever (73.5%), asthenia (64.4%), headache (54.4%), myalgia (53.3%) and back pain (50.5%). Few other signs were noticed such as: eye pain (26.7%), nausea (21.1%) and itching (14.9%) (Fig 2). Arthralgia (n = 92) was observed in several locations but most commonly in the small joints (ankles 54.3%, fingers 51.1%, hands 48.9%, feet 47.8%, knees 40.2%, wrist 25.0%, shoulders 21.7%, elbows 14.7%). The majority of the patients (73.9%) with arthralgia were affected at multiple arthralgic sites while 35.9% of these patients even complained about four or more different locations. 92.1% (58/63) of the patients reported to have difficulty with walking at onset. Regarding gender, no significant association could be identified with arthralgia at onset (women 88.1% (59/67), men 75.9% (22/29), p = 0.13).

#### Clinical course of viremic CHIKV infected patients

From day 1 to day 4, the percentage of patients with arthralgia decreased from 91.6% to 67.0%. From day 5 to day 7, a smaller steady decrease was observed (Table 2). The frequency of other symptoms decreased gradually from day 1 to day 7 (Fig 2). The occurrence of rash, however, displayed a different pattern during acute illness, with an initial increase from 19.8% on day 1 to 31.3% on day 2 and peaking on day 3 before attenuation. A similar pattern was observed for itching and nausea (Table 2).

**Table 2:** Clinical trend of arthralgia, skin rash, itching, nausea and myalgia from D0 to D7 of viremic CHIKV infected patients^a^.

Day	Arthralgia n (%)	Rash n (%)	Itching n (%)	Nausea n (%)	Myalgia n (%)
**D0**	81/96 **(84.4)**^b^	9/96 **(9.4)**	13/87 **(14.9)**	19/90 **(21.1)**	49/92 **(53.3)**
**D1**	87/95 **(91.6)**	19/96 **(19.8)**	21/87 **(24.1)**	21/89 **(23.6)**	51/92 **(55.4)**
**D2**	83/95 **(87.4)**	30/96 **(31.3)**	29/87 **(33.3)**	23/89 **(25.8)**	44/92 **(47.8)**
**D3**	75/95 **(78.9)**	36/95 **(37.9)**	28/87 **(32.2)**	25/89 **(28.1)**	36/92 **(39.1)**
**D4**	63/94 **(67.0)**	33/94 **(35.1)**	26/86 **(30.2)**	22/88 **(25.0)**	32/91 **(35.2)**
**D5**	54/90 **(60.0)**	27/88 **(30.7)**	20/81 **(24.7)**	20/83 **(24.1)**	29/88 **(33.0)**
**D6**	48/86 **(55.8)**	21/83 **(25.3)**	15/76 **(19.7)**	13/78 **(16.7)**	25/83 **(30.1)**
**D7**	44/83 **(53.0)**	21/81 **(25.9)**	15/73 **(20.5)**	10/75 **(13.3)**	24/80 **(30.0)**

^a^ n represents the actual number of patients reached by telephone for day-specific data.

^b^ Some patients had a delayed arthralgia onset: nine patients at D1, one at D3 and one at D4. The remaining four patients were included by the physician because of the presence of fever and arthralgia, however, the latter was not reported by the patients during telephone interviews.

Maculopapular skin rash was reported (n = 49) on the following sites: limbs (87.8%), trunk (63.3%) and face (10.2%), with 38.8% of these patients reporting rash over the entire body. None of the patients in this study noticed bullous skin lesions. The disease seemed to be self-limiting within 7 days for 20% (9/45) of our viremic CHIKV infected cohort.

From day 14 (median 15.0, 95% CI: 12.0–22.2) till day 90 (median 94.0, 95% CI: 79.7–114.8), a further decline was observed for all reported symptoms. Arthralgia was the most prominent clinical feature in this follow-up period (Fig 2).

Results from interviews at D180 were available for 91.8% of the individuals enrolled in the telephone survey (8 patients were lost to follow-up). From the 90 patients who could be contacted around day 180 (median 187.0, 95% CI: 181.0–210.9), twenty (22.2%) perceived an incomplete recovery and reported residual arthralgia, with 25.0% of the patients in this category reporting continuous joint pain. Persistent arthralgia was significantly more common in women (women 30.6% (19/62), men 3.7% (1/28), p = 0.004). Older age was also a significant factor for incomplete recovery (42.5 *vs* 31.0 years, median, p = 0.045). At D90, none of the patients reported a relapse, however, at D180, 8.9% of the patients reported relapses.

#### Differences between CHIKV infected children (≤12 years) and persons >12 years

As only a few studies describe clinical manifestations in children, the symptom development in children ≤12 years (categories under 5 years and schoolchildren) was compared to persons above 12 years, despite the limited numbers. We included 13 children of whom 100% were confirmed as viremic CHIKV infected (10.7% ≤12 years) and 76.9% were reached for follow-up. The registered symptoms at inclusion such as headache and rash were similar in both groups with the exception of arthralgia and vomiting (Table 3). Arthralgia at onset was reported less frequently for children, while on the other hand, vomiting was reported more frequently at onset for children (Table 3). The frequency of arthralgia reported at day 1 and day 2 was similar compared to persons above 12 years. However, between day 3 and 7, and at day 30 it was significantly lower for children (Table 3). Maculopapular rash was mostly reported at day 4 on the following sites: face 40% (2/5) and limb 60% (3/5). Children reported less frequent back pain at day 3 (Table 3). One child (an 8-years-old girl) had intermittent joint pain until 6 months after infection, while none of the children reported a relapse.

**Table 3:** Differences in clinical manifestations of viremic CHIKV infected children and adults^a^.

	Children n (%)^b^ (0–12 years)	Adults n (%) (>12 years)	p-Value
**Arthralgia**			
**D0**	5/10 **(50.0)** ^c^	76/86 **(88.4)**	0.020
**D1**	8/10 **(80.0)**	79/85 **(92.9)**	0.160
**D2**	8/10 **(80.0)**	75/85 **(88.2)**	0.460
**D3**	5/10 **(50.0)**	70/85 **(82.4)**	0.018
**D4**	3/9 **(33.3)**	60/85 **(70.6)**	0.024
**D5**	2/9 **(22.2)**	52/81 **(64.2)**	0.015
**D6**	2/9 **(22.2)**	46/77 **(59.7)**	0.032
**D7**	2/9 **(22.2)**	42/74 **(56.7)**	0.050
**D30**	1/9 **(11.1)**	49/77 **(63.6)**	0.003
**Symptoms at onset**			
**Headache**	6/7 **(85.7)**	43/83 **(51.8)**	0.084
**Rash**	0/10 **(0.0)**	9/86 **(10.5)**	0.370
**Vomiting**	2/7 **(28.6)**	3/86 **(3.5)**	0.005
**Symptoms D1-D7**			
**Back pain day 3**	1/7 **(14.3)**	46/85 **(54.1)**	0.043
**Rash day 4**	4/9 **(44.4)**	29/85 **(34.1)**	0.537

^a^ n represents the actual number of patients/parents reached by phone for day-specific data.

^b^ Arthralgia and rash were registered for all children, however, the other symptoms were registered only for children above 4 years old (n = 7).

^c^ Three children had a delayed arthralgia onset at D1. The other two children were included by the physician because of the presence of fever and arthralgia, however, the latter was not reported by the parent during telephone interviews.

### Community-based surveillance

Between October 2014 and March 2015, a total of 4842 participants (1637, 1583 and 1622 in the three surveys respectively) from 1169 households (385, 392, and 392, respectively) in all 12 regions in Paramaribo were included. The random distribution of households and the cross-sectional study design allowed inference and valid analysis.

The data presented encompass the whole study period. The gender distribution of this survey followed the general Surinamese population distribution (m/f ratio: 1.1 *vs* 1.0). The limited number of participants in each of the twelve regions in Paramaribo did not allow for comparisons per region.

#### Cumulative incidence

Suspected CHIKV was reported in 53.1% of the households, resulting in a cumulative incidence in the period July 2014 till March 2015 of 276 cases per 1000 persons. The incidence was significantly higher for women than men (328 cases/1000 *vs* 226 cases/1000, p<0.0001). The serological testing on the presence of CHIKV antibodies in a random subset of samples (n = 52) from the self-reported CHIKV positives revealed that 90.4% carried IgG antibodies, while 17.3% also displayed IgM antibodies. This high percentage validates the value of self-reporting CHIKV infection. The actual CHIKV cumulative incidence in the community-based surveillance by inference was 249 cases per 1000 persons.

#### Care seeking behavior

The investigation of the health care seeking behavior revealed that 79.5% of the CHIKV suspected participants sought medical care. We observed that CHIKV suspected women more frequently visited the physician (women 81.8% *vs* men 71.4%, p = 0.0098). The health care seeking behavior displayed some differences over time, although the number of persons seeking health care was high in all three surveys (69.6%, 65.7% and 79.5%, respectively, p = 0.0010).

#### Use of insect repellent

47.4% of the households reported use of insect repellent. The results obtained in the first and second survey for insect repellent use were similar (44.8% and 47.5%, respectively). In the first two surveys the use of insect repellent in households with self-reported CHIKV was significantly higher than households without CHIKV (survey 1: 52.1% *vs* 40.4%, p = 0.026; survey 2: 54.4% *vs* 41.3%, p = 0.010). This difference was not observed in the third survey (49.5% *vs* 45.7%, p = 0.45).

#### Trend of suspected CHIKV cases

**Fig 3:**
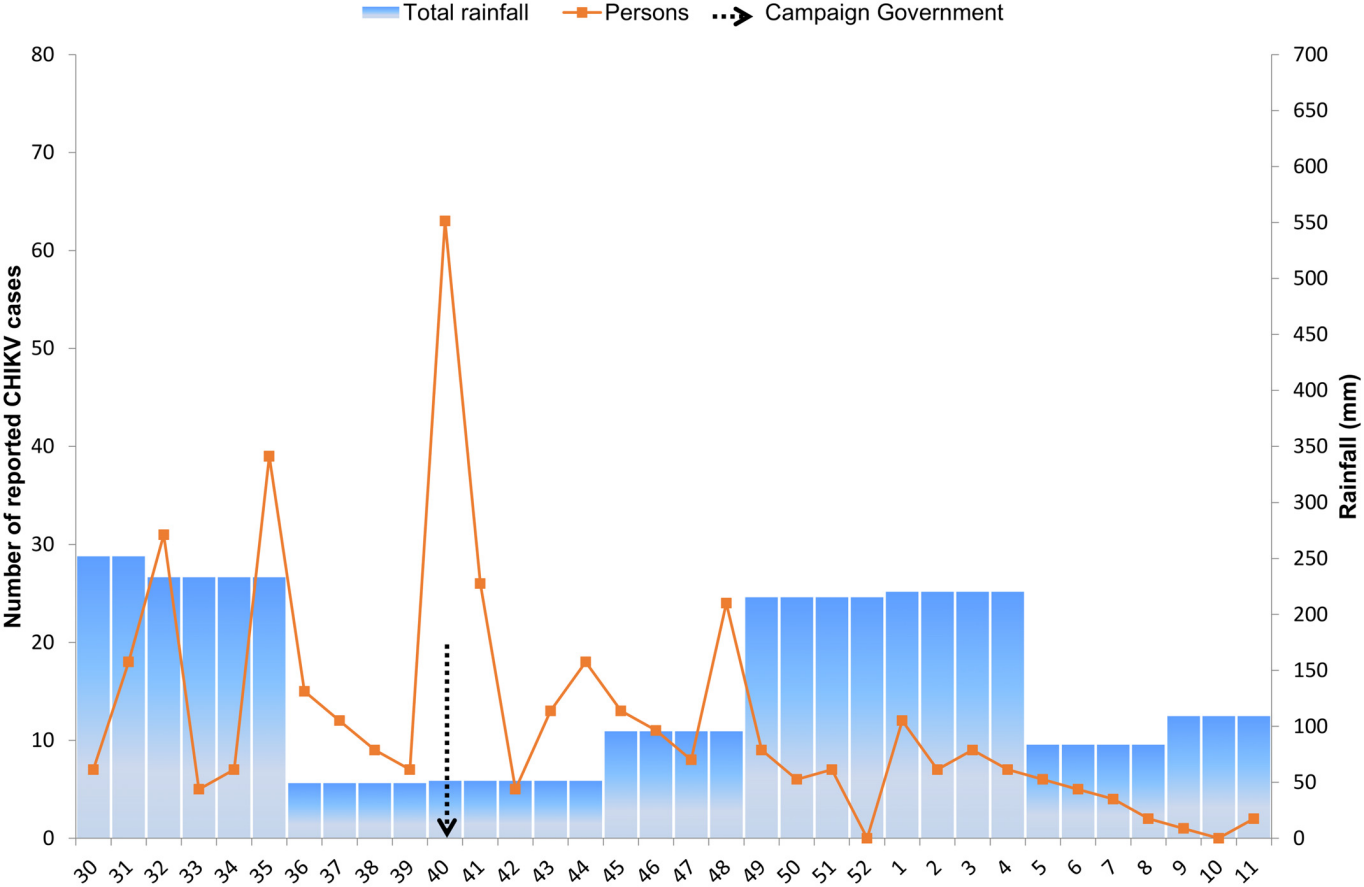
Number of reported CHIKV cases reported (July 2014-March 2015). Monthly rainfall (mm, obtained from the Meterological Center in Suriname) is also depicted; for weeks containing overlapping days from two months (*i*.*e*. week 31, 36, 40, 44 and 49), rainfall is depicted for the month with the most days in that week.

After the first confirmed case in June, the outbreak irregularly increased and peaked after 14 weeks at week 40. This sharp increase of suspected cases occurred in the long dry season (average rainfall 50 mm/month); five weeks after a period of heavy rains (average 233 mm/month). A sharp decline was noted after week 40, coinciding with a governmental campaign to reduce mosquito breeding sites. The number of suspected CHIKV cases tapered off after week 48 (Fig 3).

## Discussion

After the emergence of CHIKV infections in the Caribbean region in 2013–2014 [15], autochthonous infections were continuously reported in Suriname since June 2014, as illustrated in this study. The prospective follow-up study included 180 symptomatic patients with 68% testing positive for viral RNA (viremic CHIKV infected patients), of which 70.8% had severe joint pain. At disease onset 73.5% viremic CHIKV infected patients had fever and 84.4% had arthralgia which was still reported at D180 for 22.2%.

The combination of fever and severe arthralgia/arthritis in the clinically suspected sample population (68% RT-rPCR positive), demonstrated that with these cardinal symptoms, general practitioners in Suriname were able to correctly diagnose the majority of the patients during this CHIKV outbreak. The fact that only RT-rPCR was used to define CHIKV infected patients may have induced a classification bias, especially in the context of the Asian lineage strain, circulating in the Caribbean Region [17]. However, we still observed clear differences between viremic CHIKV infected and CHIKV- patients. The manifestation of arthralgia in the fingers was characteristic for viremic CHIKV infected patients, while CHIKV- patients had more frequent pain in the eyeball compared to viremic CHIKV infected patients, and less frequent arthralgia. Moreover, rash in CHIKV- patients was mostly observed at D5 and D6, in contrast to the viremic CHIKV infected patients with rash mostly at D3. These symptoms may point towards Dengue virus infection, a common infection in South-America. Furthermore, the CHIKV outbreak substantiates the vector presence and Dengue is clinically difficult to differentiate from Chikungunya [23]. In the clinical differentiation, the symptoms arthralgia and onset of rash may therefore be good markers to differentiate Chikungunya from other exanthematous diseases. Besides, additional laboratory tools as leukocyte and thrombocyte count could be utilized, since patients with Chikungunya often have lymphocytopenia which is seldom seen in Dengue patients [24], whereas thrombocytopenia as seen in patients with Dengue hemorrhagic fever is not common in Chikungunya.

The gathered detailed information about daily clinical symptoms of acute disease and chronic illness of viremic CHIKV infected outpatients in Suriname revealed that the most common reported symptoms were abrupt onset of fever, arthralgia, asthenia and myalgia. These findings, in particular the very high frequency of fever and the incapacitating peripheral pain in multiple smaller joints (*i*.*e*. ankles, hands, feet, knees) matched the main reported features of CHIKV outpatients and hospitalized patients in other regions [3, 7, 25, 26]. However, the frequency of pain by location (ankles 54.3%, hands 48.9%, feet 47.8%, knees 40.2%) during acute CHIKV infection was lower than those reported by the TELECHIK cohort study [27] (ankles 74.9%, hands 75.7%, feet 73.1%, knees 67.6%) and the French soldiers cohort (ankles 68%, fingers and palms 76%, feet 68%, knees 58%) [28]. This finding is consistent with the presumed circulation of the Asian lineage in Suriname, which is less virulent than the ECSA strain circulating in the latter population-based studies in La Reunion [17].

The overall assigned score of pain intensity in other studies was generally high (*i*.*e*. NRS score ≥7) [25] as was corroborated by our findings (score 8). The intense joint pain caused walking difficulties in almost all our viremic CHIKV infected patients (92.1%), which is even higher than during the outbreak in La Reunion (2005–2006), where 46.4% to 75.0% [21, 25] of the viremic and/or serologically-confirmed patients reported discomfort in performing daily activities such as walking.

The presence of periarticular edema which was described earlier [29], was also observed at the day of consultation in 25.5% of individuals positive for CHIKV-RNA in our cohort. This corresponds with a hospital-based study (periarticular edema in 25.6% RT-rPCR CHIKV+ patients) [30] and another study from La Reunion where 30% of patients presented with soft-tissue swelling [1, 7].

The presence of acute arthritis in 35.8% of our viremic CHIKV infected cohort is lower in comparison to the French soldiers cohort study from La Reunion with 44.8% reporting polyarthritis [28], as could be expected from the Asian lineage. Moreover, a study from India (Maharashtra State) even observed arthritis in 68.8% [31].

Symptoms improved gradually during the acute phase, except for skin rash and itching, consistent with previous reports [4, 32]. The presence of skin rash, in 37.9% of our patients, falls within the broad range that is reported worldwide (10% to 81%) [33, 34]. The occurrence of skin rash towards the end of the febrile phase [35] is substantiated by the peak presence on day 3 in our study. The general observations on maculopapular rash [35], mostly reported on limbs and trunk, rarely affecting the face and occasionally spreading over the entire body was corroborated by our findings.

At day 7, fever receded for most patients, but asthenia and arthralgia were still reported by more than 40% of the patients, in coherence with the study of Thiberville *et al*. [4]. This study also reported a considerable percentage of patients with headache and myalgia at day 7, in contrast to our findings.

Twenty percent of our cohort was symptom-free within 7 days after disease onset, which seems slightly more favorable than in La Reunion with a duration of symptoms <15 days for 23% of the cases [25].

The manifestation and the course of CHIKV symptoms are variable and depend on several factors such as virus strain, age, gender, immune status [32, 36, 37] and possibly also on the genetic predisposition [38]. The latter is supported by the finding that Maroons were underrepresented in our study. This corresponds with a recent study where hospitalizations due to Dengue, which is transmitted by the same vector as Chikungunya, occurred least in the Maroons [39]. However, the underrepresentation of Maroons in our study could also be due to either the low population density of this ethnic group in at least one of our study sites (Commewijne) or other factors as differences in health care access.

Earlier studies report that women are more prone to CHIKV infection [31, 33, 40, 41], consistent with our results, probably because of greater home-based activities and different clothing behaviors enabling an increased accessibility for mosquitoes. This is further supported by a report in the Midwest region of Brazil, where women were significantly more frequent victims of Dengue [42]. On the other hand, a cross-sectional study in Mayotte (Indian Ocean), found that CHIKV seroprevalence was higher in men than women [43] and during an outbreak in Italy no difference in sex incidence was noticed [12]. The inconsistency of sex as factor in exposure to viral infection across countries/communities may be related to different lifestyles and behaviors.

The occurrence of Chikungunya viral infections in Suriname in all age groups substantiates the general finding. Moreover, the observation that children were the least affected group during first epidemic periods in La Reunion (18% of the cases <10 years) and India (Maharashtra State: 5% of the cases <15 years) [31, 41] was also corroborated with our findings (10.7% ≤12 years). In line with recent studies [44], arthralgia (50% at onset) had a milder course in the children younger than 13 years and back pain was less common (14.3% at D3). Most studies report a low frequency of headache in children (15% to 35.3%) [44, 45], whereas a higher frequency of headache for children was reported by our cohort (85.7% at onset). The frequency of vomiting (28.6% at D0) falls within the reported range from studies in India (12.2% to 47%) [33, 45]. However, these studies did not report our observation of a significant higher frequency of vomiting for children compared to adults at onset. This discrepancy could be due to different study settings. The presence of maculopapular rash in 44.4% of the children at D4 was in line with earlier reports (33–60%) [44]. However, the earlier reported more prominent presence of skin rash in children versus adults during a CHIKV outbreak in southern Thailand [24], could not be substantiated in this study. The standard triad of fever, joint pains and rashes for the diagnosis of Chikungunya in children may not be as effective in Suriname. The low number of children included in our study however could obscure this presumption.

Persistent arthralgia was described in La Reunion and Italy in more than half of the Chikungunya patients in outpatients and hospitalized patients [21, 25, 46], while in our setting, arthralgia was observed in 22% of the patients six months after disease onset. In contrast to our study, patients in the aforementioned studies reported underlying osteoarthritis or pre-existing rheumatic diseases, which are independent markers for persistent pain [21, 46]. A recent retrospective study from Columbia also reported a higher percentage of persistent polyarthralgia (44.3%) [47]. Our results are more in line with another study in Reunion Island including only outpatients, where the residual arthralgia was significantly lower (23%) after 300 days [4]. Our results are also consistent with a study done in Indonesia, where CHIKV infections by the Asian genotype caused mild and short lasting clinical symptoms [48]. An even lower percentage of only 13.3% of residual arthralgia, 2 to 3 months after illness, was observed in a study in Singapore, with patients without underlying medical conditions [32].

Other variables associated with persistent joint pain are age ≥ 45 years and gender (women are more likely to have persistent arthralgia) [4, 25, 26, 49], as was corroborated by our results.

The frequency of patients with continuous pain (25.0%) was lower than reported by the French soldiers cohort (41.3%) [28]. Moreover, in this cohort 93.7% of the symptomatic patients still complained about chronic pains 6 months after infection (our study: 22%). This difference could be caused by the less pathological outcome of the Asian lineage presumably present in Suriname compared to the Indian Ocean strain [17].

The reported relapse of arthralgia in some Chikungunya patients in the months after acute infection [21, 23], was also observed in our study (8.9%). However, relapse was less common in Suriname than in the hospitalised-based study from Borgherini *et al*. (21%) [21]. Moreover, in the population-based French soldier cohort, the more virulent ECSA strain caused a relapse in 58.7% of the patients [28].

Our investigation of the clinical manifestations had some limitations. Firstly, we only performed qualitative PCR analysis and did not determine the average viral load at inclusion of the study. We could therefore not relate viral load to symptomatology and severity of symptoms. Secondly, clinical symptoms were only described for Chikungunya infections in patients consulting a physician in the initial stage of disease. The described symptoms are therefore expected to be more severe than the general symptoms, since less severe or asymptomatic cases were not included. Thirdly, symptomatic differences between viremic CHIKV infected and CHIKV- patients could be obscured, since symptoms of CHIKV- patients were characterized only in the acute phase. Furthermore, no serology was performed to differentiate between real negative CHIKV cases and potential CHIKV+ cases in a late stage with no detectable viremia. These reasons may have skewed the estimation of some symptoms in this cohort. However, because of the robust size of the viremic CHIKV infected sample (2 fold higher than the CHIKV- group), we feel that our conclusions about the viremic CHIKV infected outcome and symptoms are well supported.

The prospective follow-up study was complemented with cluster-based cross-sectional data and the cumulative incidence in Paramaribo from July 2014 to March 2015 was 249 cases per 1000, which by inference is similar to the cumulative incidence of La Reunion in 2005/2006 (350 cases per 1000) over a 12-month period [41]. Caution is warranted in the country comparison for epidemic impact, since several factors as virus strain, influence of the weather, the density of the population and the effectiveness of vector control measures are involved.

The high value of serologically confirmed CHIKV infections (90.4% IgG) in self-reported cases clearly demonstrated the acquired skill of the population to recognize and diagnose CHIKV infection.

Internationally, not much data is available about the health care seeking behavior during Chikungunya outbreaks. In Mayotte, 52% of participants with confirmed CHIKV sought medical advice [50], while 79.5% of the CHIKV suspected persons in our study population sought medical care. The good access to health care in Paramaribo may account for this high percentage.

In the community surveillance, more women self-reported to be Chikungunya positive, underlining the observation among the laboratory confirmed CHIKV cases that women are more prone to CHIKV infection. Moreover, also the health care seeking behavior was higher for women than men, corroborating the results of a general study on the utilization of health care services in the USA [51] and underlining the generally poor recovery observed in women.

The use of insect repellent (47.4%) in Paramaribo is lower than in outbreaks in La Reunion (67.9%) [52] and India (Chennai) (88.4%) [53], despite the fact that in contrast to Reunion Island, Suriname was warned for an upcoming Chikungunya outbreak. However, the latter two studies reported individual use of insect repellent while in our study household data were gathered. Moreover, country comparisons may not be very useful without data of the mosquito index and data on actual repellent use.

In Suriname, the highest CHIKV incidence was noted in the long dry season, which may have been associated with increased storage of water filled objects in dry periods, positively influencing mosquito breeding and thus CHIKV transmission. This premise is further supported by the observation that the sharp increase in CHIKV cases occurred five weeks after the dry season had set in, similar to the largest Dengue epidemic in Suriname in 2005 (personal communication) and in coherence with findings in Thailand, where Chikungunya peaked in several provinces 6 weeks after the start of the dry period [54].

The sharp decline of CHIKV after week 40, coincided with the start of the governmental campaign to eliminate mosquito breeding sites, which may have positively impacted the containment of this outbreak. These results thus support country actions to increase effectiveness of vector control and to raise population awareness.

In conclusion, this is the first report about the emergence of CHIKV in Suriname. The clinical course of most symptoms in our naïve cohort was similar to those of other countries that faced a Chikungunya outbreak. The earlier finding that women and elderly persons are more at risk for persistent arthralgia was substantiated, although our study highlighted that persistent arthralgia, mostly intermittent, is a less frequent concern among viremic CHIKV infected patients in an outpatient setting. Furthermore, our data support the presumption of the circulation of the Asian lineage of CHIKV in Suriname. Our findings also provided more insight into the manifestation and course of this arboviral disease in children. Still, data from larger children cohorts in CHIKV affected areas are required to further enhance patient management. We also observed some clinical differences between viremic CHIKV infected and CHIKV- patients, which could be useful to differentiate Chikungunya from other diseases (such as exanthematous diseases as Dengue) in resource-limited countries lacking testing facilities. The finding that 20% of the patients cleared the disease within one week, while 22% still displayed persistent arthralgia after six months offers valuable indicators for countries facing their first outbreak. Furthermore, our study adds to the international data on cumulative Chikungunya incidence during first outbreaks, which is particularly important for the Caribbean region.

## Supporting Information

S1 Checklist STROBE checklist. (PDF)
